# Polymorphisms of the endothelial nitric oxide synthase (*NOS3*) gene in preeclampsia: a candidate-gene association study

**DOI:** 10.1186/1471-2393-11-89

**Published:** 2011-11-03

**Authors:** Nikos Zdoukopoulos, Chrysa Doxani, Ioannis E Messinis, Ioannis Stefanidis, Elias Zintzaras

**Affiliations:** 1 Department of Obstetrics and Gynaecology, University of Thessaly School of Medicine, Larissa, Greece; 2 Department of Biomathematics, University of Thessaly School of Medicine, Larissa, Greece; 3 Department of Nephrology, University of Thessaly School of Medicine, Larissa, Greece; 4 Center for Clinical Evidence Synthesis, The Institute for Clinical Research and Health Policy Studies, Tufts Medical Center, Tufts University School of Medicine, Boston, MA, USA

## Abstract

**Background:**

The endothelial nitric oxide synthase gene (*NOS3*) has been proposed as a candidate gene for preeclampsia. However, studies so far have produced conflicting results. This study examines the specific role of variants and haplotypes of the *NOS3 *gene in a population of Caucasian origin.

**Methods:**

We examined the association of three common variants of the *NOS3 *gene (4b/a, T-786C and G894T) and their haplotypes in a case-control sample of 102 patients with preeclampsia and 176 women with a history of uncomplicated pregnancies. Genotyping for the *NOS3 *variants was performed and odds ratios and 95% confidence intervals were obtained to evaluate the association between *NOS3 *polymorphisms and preeclampsia.

**Results:**

The single locus analysis for the three variants using various genetic models and a model-free approach revealed no significant association in relation to clinical status. The analysis of haplotypes also showed lack of significant association.

**Conclusions:**

Given the limitations of the candidate-gene approach in investigating complex traits, the evidence of our study does not support the major contributory role of these common *NOS3 *variants in preeclampsia. Future larger studies may help in elucidating the genetics of preeclampsia further.

## Background

Preeclampsia is a medical condition in which high blood pressure and elevated urinary excretion of protein develop in pregnancy [1]. Family-based studies have shown that genetic factors may play a role in preeclampsia [2]. In addition, candidate-gene association studies (GAS) on preeclampsia have not produced conclusive results so far [3]. However, the pathogenesis of preeclampsia is poorly understood and the search for low-penetrance genes by hypothesis-driven candidate-gene studies (genetic association study-GAS) and hypothesis-free genome-wide association studies is ongoing [4].

The leading hypotheses, concerning the pathogenesis of preeclampsia, are based on disturbed placental function and impaired remodelling of the spiral arteries [5]. Endothelial nitric oxide synthase (NOS3) is an important regulator of vascular tone and contributes to the reduction of the uteroplacental resistance seen in normal pregnancy [6-8]. Therefore, the endothelial nitric oxide synthase gene (*NOS3*), located at the 7q35-q36 region, has emerged as a logical candidate gene in the development of preeclampsia. Variants (polymorphisms) of the *NOS3 *gene have been investigated for association with preeclampsia and other disorders such as hypertension [9,10]. The three most common variants examined for clinical relevance, based on their potential functional effects are [11]: (i) a G894T substitution in exon 7 resulting in a Glu to Asp substitution at codon 298 (rs1799983), (ii) an insertion-deletion in intron 4 (4a/b) consisting of two alleles (the a*-deletion which has four tandem 27-bp repeats and the b*-insertion having five repeats), and (iii) a T786C substitution in the promoter region (rs2070744). A whole genome-scan meta-analysis for preeclampsia has already identified the locus of *NOS3 *gene as a promising candidate for preeclampsia susceptibility [2], although linkage studies seem to support a relationship between *NOS3 *and hypertension rather than preeclampsia [12,13].

Previous GAS that investigated the association between *NOS3 *variants and preeclampsia have produced controversial or inconclusive results and the replication record of these studies is relatively poor [14,15]. Therefore, the status of association for the *NOS3 *variants remains ambiguous. In this project we aimed to replicate previous findings on the association between the three most commonly investigated *NOS3 *polymorphisms (4b/a, T-786C and G894T) and preeclampsia, in a GAS conducted on a homogeneous population of Caucasian origin (Greeks). These variants were chosen because of their potential functional implications and their high minor allele frequency [16-18]. An analysis of haplotypes was also performed [19].

## Methods

### Study population

A total of 102 cases with preeclampsia and 176 female controls were recruited from the Department of Obstetrics and Gynecology, University Hospital of Larissa. Preeclampsia was defined as new hypertension (systolic blood pressure ≥140 mmHg or diastolic blood pressure ≥90 mmHg) and substantial proteinuria (>300 mg in 24 h) at or after 20 weeks' gestation [20]. The study protocol was approved by the Institute Ethics Committee and all subjects provided a written informed consent. All controls faced pregnancies without complications and they were free of preeclampsia history. The controls were unrelated by blood to cases, and all apart from 3 (1.7%) were delivered at term. All study participants were of Caucasian origin whereas women with previous renal disease, diabetes, or history of metabolic disorders were excluded from the patient sample. A blood sample for biochemical measurements and DNA extraction was taken from each individual.

### Laboratory assays

Genomic DNA was extracted from whole blood using the QIAamp DNA blood kit (QIAGEN, Valencia, CA, USA) following the manufacturer's instruction. Genotyping of each polymorphism was performed by amplification from 50 to 100 ng of genomic DNA. The primer sequences used and the laboratory conditions for genotyping (polymerase chain reaction, restriction enzymes, agarose electrophoresis) for each *NOS3 *polymorphism have been previously described [16-18]. Genotyping was performed by laboratory personnel blinded to clinical status.

### Data Analysis

Continues variables were compared using the Mann-Whitney non-parametric test. Categorical variables were compared using the Chi-square test. The genotypic distribution in the control group was tested for departing from the Hardy-Weinberg Equilibrium (HWE) and the existence of linkage disequilibrium (LD) between the two polymorphisms in both cases and control was tested by using exact tests according to Weir [21]. The haplotype frequencies were estimated and compared using SHEsis [22]. The association between genotype distribution and clinical status was tested using the chi-square test. The dominant and recessive models of the cases was compared to the control group using a logistic model. The significance of the genetic models was also tested using the Fisher's exact test. The comparisons associations were expressed in terms of odds ratio (OR), unadjusted and adjusted for age, with the corresponding 95% confidence interval (CI). The statistical analysis was performed using SPSS v11.5 (SPSS Inc.). A result was considered statistically significant when P < 0.05.

## Results

A total of 102 cases and 176 controls were analyzed. The mean age (±SD) was 30.64 ± 6.32 and 29.60 ± 5.18 for cases and controls respectively; all subjects were females. The clinical characteristics of the enrolled subjects are shown in Table 1. The distributions of the *NOS3 *4b/a, T-786C and G894T genotypes were not associated with clinical status and are presented in Table 2. The generalized ORs for 4b/a, T-786C and G894T polymorphisms were ORG = 0.79 (0.48-1.29), ORG = 0.93 (0.61-1.43) and ORG = 0.86 (0.56-1.32), respectively. The genotype distribution in controls was in HWE for all polymorphisms, indicating lack of stratification and/or genotyping error (p = 0.99 for 4b/a, p = 0.99 for T-786C and p = 0.090 for G894T).

**Table 1:** Characteristics of the study subjects.

	Cases	Controls	P-value
*N*	102	176	

Age (years)	30.64 ± 6.32	29.60 ± 5.18	0.14

Delivery weeks	35.41 ± 3.43	38.58 ± 2.14	<0.01

Birth weight (g)	2266.5 ± 839	3078.0 ± 572	<0.01

First parity	74 (72.5%)	78 (44.3%)	<0.01

Systolic BP	161.66 ± 10.3	115.84 ± 10.9	<0.01

Diastolic BP	92.70 ± 8.47	70.40 ± 7.54	<0.01

Smoking before/ during pregnancy	30 (29.4%)/ 6 (5.9%)	62 (35.2%)/ 16 (9.1%)	0.32/ 0.34

Twin pregnancies	8 (7.8%)	3 (1.7%)	0.01

**Table 2:** Distribution (%) of the *NOS3 *4b/a, T-786C and G894T genotypes among cases and control subjects.

SNP	Genotype	Cases (n = 102)	Controls (n = 176)	p-value
4b/a	bb	66.7	61.9	0.316 ORG = 0.79 (0.48-1.29)
	ab	32.4	34.1	
	aa	0.98	3.98	

T-786C	CC	13.7	15.9	0.868 ORG = 0.93 (0.61-1.43)
	TC	50	47.7	
	TT	36.3	35.2	

G894T	TT	16.7	13.6	0.237 ORG = 0.86 (0.56-1.32)
	GT	37.3	47.7	
	GG	46.1	38.6	

The p-values and the generalized odds ratios (ORG) with their respective 95% confidence intervals for testing the association between genotype distribution of each polymorphism and susceptibility to preeclampsia are shown.

Table 3 shows the association results for the allele contrast, the recessive and dominant models for the mutant alleles. None of the polymorphism showed significant association with development of preeclampsia for allele contrast, the recessive and dominant models. The unadjusted ORs for the allele contrast were OR = 0.78 (0.50-1.21) for 4b/a, OR = 0.94 (0.66-1.34) for T-786C and OR = 0.91 (0.65-1.30) for G894T. The age-adjusted ORs produced similar results.

**Table 3:** Association results.

SNP	Genetic contrast	OR (95% CI)	p-value	OR_adjusted _(95% CI)
4b/a	Allele contrast (b vs. a)	0.78 (0.50-1.21)	0.27	
	Recessive model	0.81 (0.49-1.36)	0.43	0.81 (0.48-1.35)
	
	Dominant model	0.24 (0.03-1.97)	0.18	0.24 (0.03-1.97)

T-786C	Allele contrast (C vs. T)	0.94 (0.66-1.34)	0.73	
	
	Recessive model	0.83 (0.41-1.17)	0.59	0.81 (0.40-1.63)
	
	Dominant model	0.97 (0.59-1.62)	0.91	0.93 (0.56-1.56)

G894T	Allele contrast (T vs. G)	0.91 (0.65-1.30)	0.60	
	Recessive model	1.27 (0.65-2.49)	0.49	1.25 (0.63-2.46)
	
	Dominant model	0.74 (0.45-1.21)	0.23	0.72 (0.439-1.19)

Odds Ratio (OR) and the corresponding 95% Confidence Intervals (CIs) for testing the association between preeclampsia and *NOS3 *4b/a, T-786C and G894T polymorphisms for the allele contrast, the dominant and recessive models. The ORs adjusted for age are also shown.

The LD results are shown id Table 4. In both the patient and control populations, the 4b/a and T-786C polymorphisms were in LD (p = 0.02 and p < 0.01, respectively). In addition, the T-786C and G894T polymorphisms were in LD (p < 0.01 for both cases and controls). The distribution of the estimated haplotype distribution of the two *NOS3 *polymorphisms for cases and controls is presented in Table 5. The estimated haplotype frequencies were similar in cases and controls. The global test of association was not significant for the comparison of haplotypes between cases and controls (p = 0.71).

**Table 4:** D', r^2 ^and p-values for testing linkage disequilibrium between pairs of SNPs for cases and controls (in brackets).

	T-786C	G894T
4b/a	D' = 0.66 (0.63) r2 = 0.14 (0.15) p = 0.02 (<0.01)	D' = 0.59 (0.54) r2 = 0.04 (0.05) p = 0.10 (0.11)

T-786C	-	D' = 0.45 (0.38) r2 = 0.17 (0.13) p < 0.01 (<0.01)

**Table 5:** Estimated haplotype frequencies for the three *NOS3 *polymorphisms (4b/a, T786C and G894T).

Haplotype	Haplotype frequency			
**4b/a-T786C-G894T**	**Cases**	**Controls**	**p-value**	**p-value** **overall**

a-C-G	0.13	0.14	0.59	0.71
a-T-G	0.03	0.04	0.45	
4b-C-T	0.22	0.21	0.84	
4b-T-G	0.47	0.42	0.28	
4b-T-T	0.11	0.13	0.37	

The p-values for comparing each haplotype between cases and controls, and the p-value for the overall comparison of haplotypes between cases and controls are shown.

## Discussion

This study evaluated relations between common genetic variants and haplotypes in the *NOS3 *gene with preeclampsia. The single locus analysis among the three most commonly studied polymorphisms (4b/a, T-786C and G894T) revealed lack of association. The analysis of haplotypes also showed non-significant association.

The conclusion reached in the GAS was based on relatively small number of participants for each gene polymorphism and therefore, any inferences have to be cautious. Candidate-gene studies have a tendency to lack the power to detect weak genetic contributions of common variants [19,23]. For example, in order to achieve a power >80% to identify a modest genetic effect (odds ratio 1.2) of a polymorphism present in 10% of individuals, a sample size of 10,000 subjects or more would be needed [23]. Therefore, the sample sizes required to predict association have to be far beyond what is currently available and no single institution or entity alone will be able to provide a reasonable number of patients. Meta-analysis of multiple studies has clearly a role in offering an analysis with the potential for enhanced power [19].

It has been reported that the polymorphisms might be in linkage disequilibrium and interaction of the polymorphisms within haplotypes can be a major determinant of disease susceptibility than the individual polymorphism [11]. Thus, individual *NOS3 *genotypes might not be reliable markers of risk for developing preeclampsia. The haplotype analysis approach is expected to be more powerful than single-marker analysis, because of the ancestral structure incarcerated in the distribution of haplotypes [10]. Particularly if the markers used define mutations within functional DNA, then the haplotypes composed of these markers can have more of a biological role. In our analysis, we focused on haplotypes defined by the three most commonly investigated and potentially functional *NOS3 *variants (instead of other tested variants or HapMap tagging polymorphisms) because we aimed to replicate the already examined associations [9,24,25].

Interestingly, 13 studies [9,24-35] have reported positive association for the variants *NOS3 *gene, whereas the two published meta-analyses and other individual studies did not replicate the findings [14,15,36,37]. Thus, the available evidence from candidate-gene approach cannot support a major contributory role of *NOS3 *variants in preeclampsia pathogenesis. Since predicting the functional credentials of most genetic variants remains problematic, obtaining robust replication of positive association findings proves difficult [38]. Moreover, the multifactorial etiology of common disorders involving complex epistatic and gene-environment interactions reduces the likelihood that a single type of studies, such as gene-candidate association studies, could provide conclusive inferences. An integration approach combining different kinds of genetic data analysis (i.e. genomic convergence of GAS, GWAS, genome-scans and microarrays) may be more effective in identifying valid genetic loci [39,40].

In conclusion, the genetic association study did not replicate a significant association for the *NOS3 *variants and haplotypes. The results of the present individual GAS should be interpreted with caution since the number of participants were relatively small. However, preeclampsia is a complex disease with multifactorial etiology and therefore, a minor contributory pathogenetic role of *NOS3 *variants in conjunction with other genetic or environmental factors cannot be excluded [41]. Collaborative research in preeclampsia may help in identifying the contributing role of variants by performing GAS and genome-wide association studies with adequate power [42]. Furthermore, the design of rigorous studies for investigating epistatic and gene-environment interactions and the utilization of data generated by genomic studies may help in deriving more conclusive claims about the genetics of preeclampsia.

## Conclusions

The single locus analysis and the analysis of haplotypes failed to demonstrate a significant association between preeclampsia and three common *NOS3 *polymorphisms.

## Competing interests

The authors declare that they have no competing interests.

## Authors' contributions

NZ and CD carried out the samples processing and the molecular genetic studies. NZ was involved in data acquisition, analysis and interpretation, and helped in the preparation of the manuscript. IEM participated in the design and coordination of the study and reviewed the manuscript. IS was involved in the design and coordination of the study. EZ conceived of the study, participated in its design, performed the statistical analysis and drafted the manuscript. All authors read and approved the final manuscript.

## Pre-publication history

The pre-publication history for this paper can be accessed here:

http://www.biomedcentral.com/1471-2393/11/89/prepub
